# Supplementary material for: Trabectedin derails transcription-coupled nucleotide excision repair to induce DNA breaks in highly transcribed genes
Source: Nat Commun. 2024 Feb 15;15:1388. doi: 10.1038/s41467-024-45664-7 (PMC10869700; doi:10.1038/s41467-024-45664-7)
Supplement: Supplementary file 1 — Supplementary Information [file 41467_2024_45664_MOESM1_ESM.pdf]

SUPPLEMENTARY INFORMATION FOR

Trabectedin derails transcription-coupled nucleotide excision repair to induce DNA breaks in highly transcribed genes

Kook Son[1#], Vakil Takhaveev[2#], Visesato Mor[1], Hobin Yu[1,3], Emma Dillier[2], Nicola Zilio[4], Nikolai J.L. Püllen[2], Dmitri Ivanov[1], Helle D. Ulrich[4], Shana J. Sturla[2*], Orlando D. Schärer[1,3*]

[1]Center for Genomic Integrity, Institute for Basic Science (IBS), 44919 Ulsan, Republic of Korea

[2]Department of Health Sciences and Technology, ETH Zürich, 8092 Zürich, Switzerland

[3]Department of Biological Sciences, Ulsan National Institute of Science and Technology (UNIST), 44919 Ulsan, Republic of Korea

[4]Institute of Molecular Biology (IMB), 55128 Mainz, Germany

[#]The first two authors contributed equally and are listed in alphabetical order

[*]Corresponding Authors:

Orlando D. Schärer, E-mail: orlando.scharer@ibs.re.kr

Shana Sturla, E-mail: sturlas@ethz.ch

This PDF file includes:

Supplementary Figures 1–8

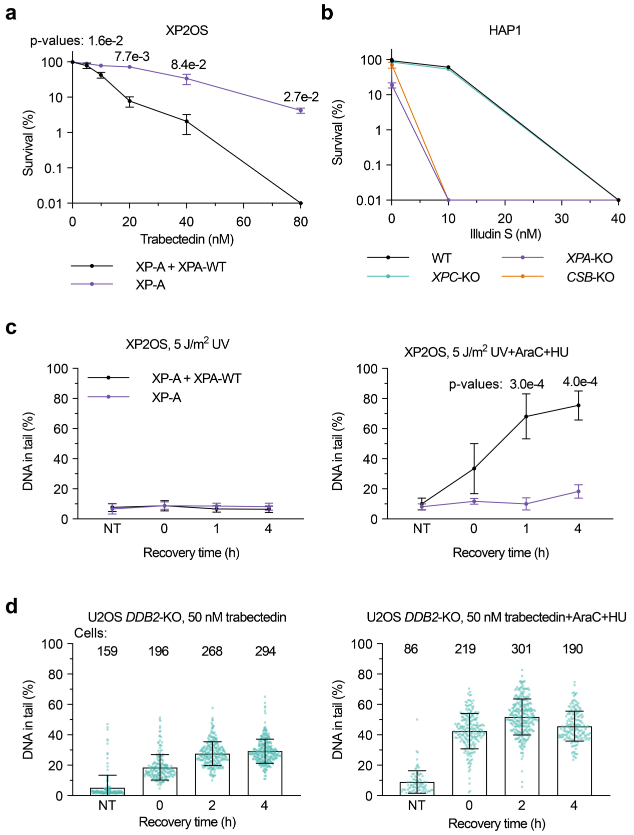

Supplementary Fig. 1, Related to Fig. 1: Trabectedin induces TC-NER-dependent DNA strand breaks in G1 cells. (a) XP2OS (XP-A or lentiviral expression of XPA-WT) cells were treated with trabectedin or DMSO for 2 h and incubated in fresh growth medium. Colony counting was done after 8 days. Shown data: mean ± SEM of n=3 biological replicates (3 technical replicates per experiment). P-values of two-tailed paired t-test (between XP-A+XPA-WT and XP-A at each concentration) are provided. **(b)** HAP1 WT, XPC-, XPA-, and CSB-KO were treated with 0.1, 10, and 40 nM illudin S or DMSO for 2 h and incubated in fresh growth medium. Colony counting was done after 8 days. Shown data: mean ± SEM of n=2 biological replicates (3 technical replicates per experiment). **(c)** XP2OS cells were treated with UV (5 J/m^2) and incubated for 1 or 4 h with or without repair synthesis inhibitors (4 mM HU, 40 mM AraC). ssDNA breaks were analyzed by alkaline COMET chip assays. Shown data: mean ± SEM of 3 biological replicates. P-values of ordinary two-way ANOVA with uncorrected Fisher's LSD (between XP-A+XPA-WT and XP-A at each recovery time) are provided. **(d)** U2OS DDB2 KO cells were arrested in G1 with palbociclib (1 µM, 24 h) and treated with trabectedin (50nM, 2 h). Cells were kept in G1 and allowed to recover for up to 4 h with or without the repair synthesis inhibitors (1 mM HU, 10 mM AraC). ssDNA breaks were analyzed by alkaline COMET chip assays. Each dot represents DNA in tail (%) of a comet analyzed. Each box represents the mean value of DNA in tail (%) from all comets used in all experiments. The number of comets used is provided above each box. An error bar represents SD. Source data are provided as a Source Data file.

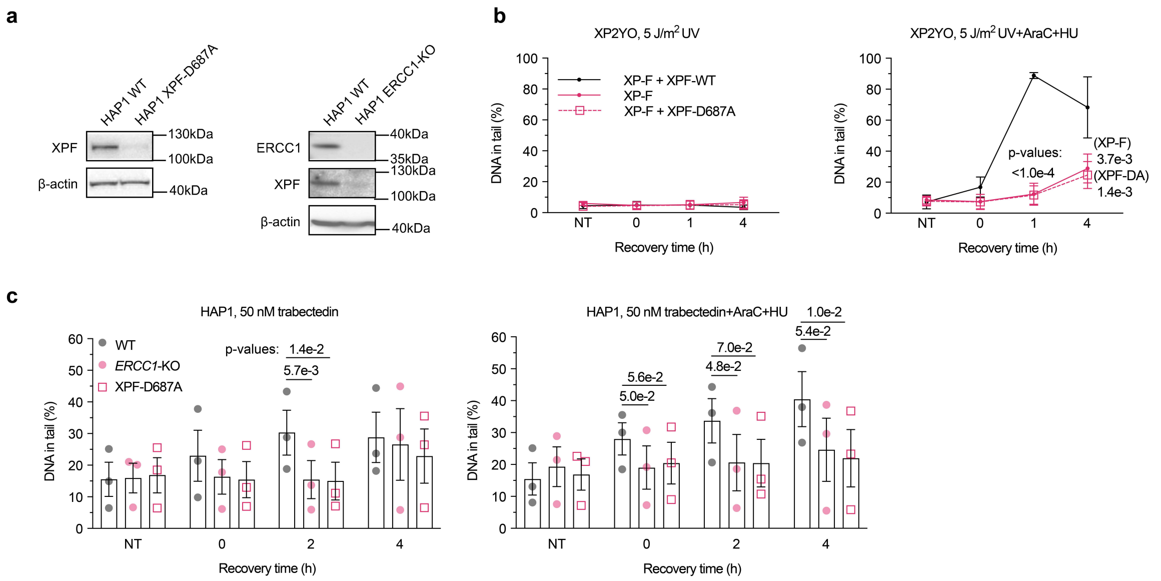

Supplementary Fig. 2, Related to Fig. 2: Trabectedin-induced DNA break formation and toxicity depend on the catalytic activity of XPF but not that of XPG. (a) Cells were lysed and subjected to western blot analysis for XPF and ERCC1 expression (n=3). **(b)** XP2YO patient cells (XP-F, with or without lentiviral expression of XPF-WT or XPF-D687A) were exposed to 5 J/m^2 UV-C and allowed to repair for 1 or 4 h with or without repair synthesis inhibitors (4 mM HU, 40 mM AraC). ssDNA breaks were analyzed by alkaline COMET chip assays. Shown data: mean ± SEM of n=3 biological replicates. P-values of ordinary two-way ANOVA with Dunnett's multiple comparisons test (between XP-F+XPF-WT and XP-F or XP-F+XPF-D687A at each recovery time) are provided. **(c) Fig.2b** in dot plots. Each dot represents the mean value of DNA in tail (%) from all comets used in an individual experiment. Each box represents the mean value of DNA in tail (%) from all comets used in all experiments. An error bar represents SEM. P-values of two-tailed paired t-test (between WT and ERCC1-KO or XPF-D687A at each recovery time) are provided. Source data are provided as a Source Data file.

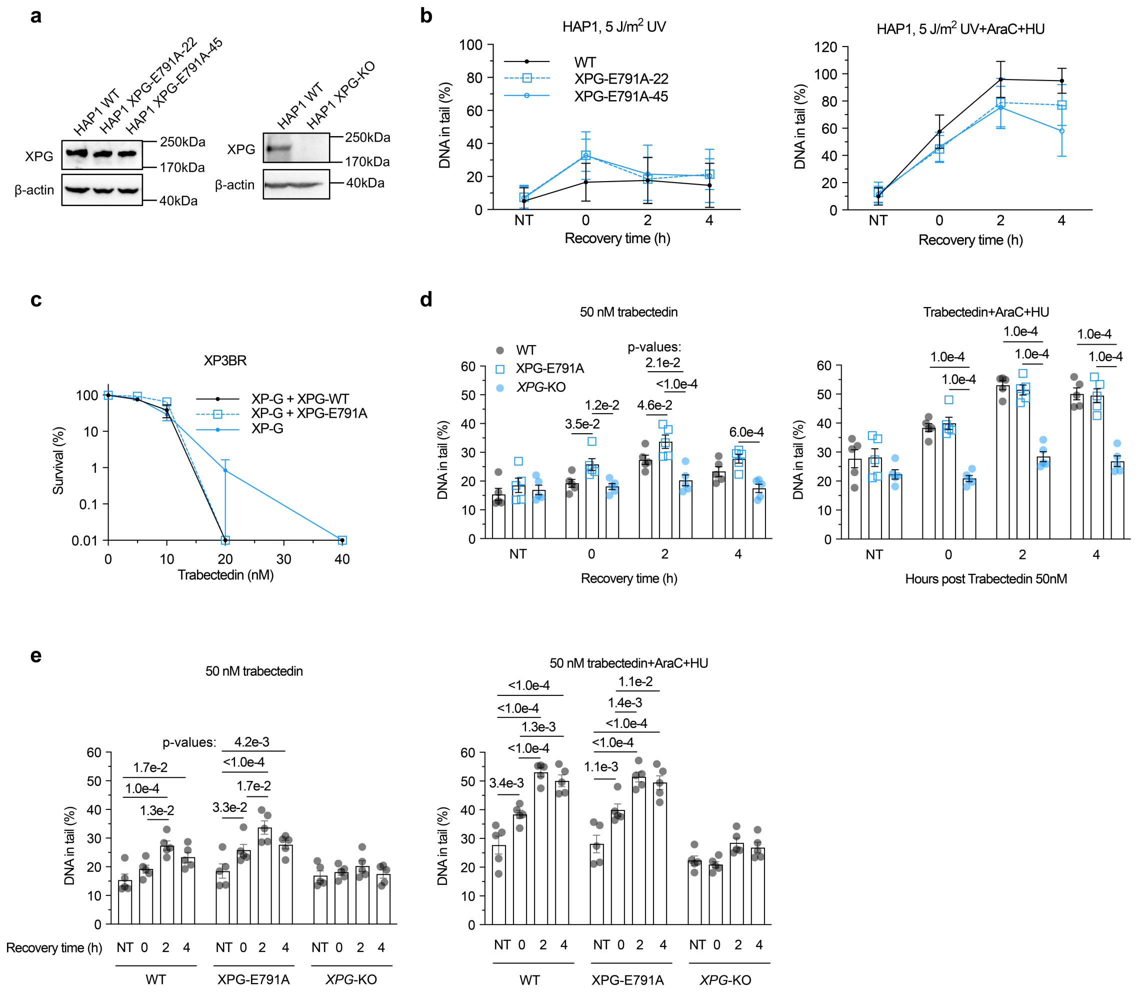

Supplementary Fig. 3. Related to Fig. 2: Trabectedin-induced DNA break formation and toxicity depend on the catalytic activity of XPF but not that of XPG. (a) Cells were lysed and subjected to western blot analysis for XPG expression (n=3). **(b)** HAP1 WT, XPG-E791A clones #22 and #45 were arrested in G1 with palbociclib (2 µM, 24 h), exposed to 5 J/m^2 UV-C and allowed to repair for up to 4 h with or without repair synthesis inhibitors (0.5 mM HU, 5 mM AraC). ssDNA breaks were analyzed by alkaline COMET chip assay (n=1). Error bars represent the polydispersity of the individual comets. **(c)** XP3BR patient cells (XP-G, with or without lentiviral expression of XPG-WT or XPG-E791A) were treated with trabectedin or DMSO for 2 h and incubated in fresh growth medium. Colony counting was done after 8 days. Shown data: mean ± SEM of n=2 biological replicates (3 technical replicates per experiment). **(d-e) Fig. 2f** in dot plots. Each dot represents the mean value of DNA in tail (%) from all comets used in an individual experiment. Each box represents the mean value of DNA in tail (%) from all comets used in all experiments. An error bar represents SEM. P-values of ordinary two-way ANOVA with Tukey's multiple comparisons test are provided. Comparison of WT, XPG-E791A, and XPG-KO at each recovery time (**d**); and between the recovery time for the individual cell lines (**e**). Source data are provided as a Source Data file.

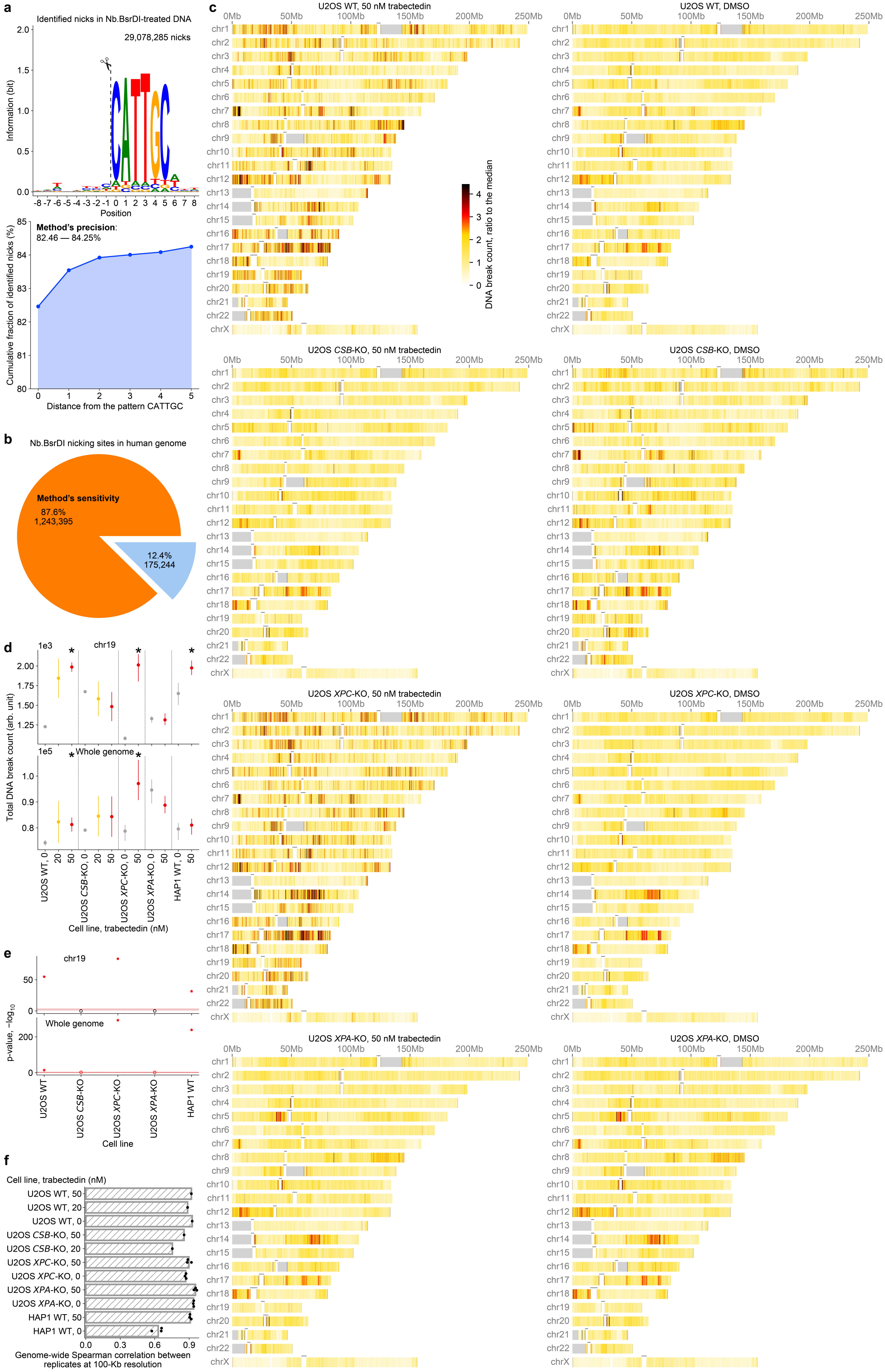
a
Identified nicks in Nb.BsrDI-treated DNA
29,078,285 nicks
Information (bit)
Position
Method's precision:
82.46 — 84.25%
Cumulative fraction of identified nicks (%)
Distance from the pattern CATTGC
b
Nb.BsrDI nicking sites in human genome
Method's sensitivity
87.6%
1,243,395
12.4%
175,244
c
U2OS WT, 50 nM trabectedin
U2OS WT, DMSO
U2OS CSB-KO, 50 nM trabectedin
U2OS CSB-KO, DMSO
U2OS XPC-KO, 50 nM trabectedin
U2OS XPC-KO, DMSO
U2OS XPA-KO, 50 nM trabectedin
U2OS XPA-KO, DMSO
DNA break count, ratio to the median
d
chr19
Whole genome
Total DNA break count (arb. unit)
Cell line, trabectedin (nM)
e
chr19
Whole genome
p-value, −log10
Cell line
f
Cell line, trabectedin (nM)
U2OS WT, 50
U2OS WT, 20
U2OS WT, 0
U2OS CSB-KO, 50
U2OS CSB-KO, 20
U2OS XPC-KO, 50
U2OS XPC-KO, 0
U2OS XPA-KO, 50
U2OS XPA-KO, 0
HAP1 WT, 50
HAP1 WT, 0
Genome-wide Spearman correlation between replicates at 100-Kb resolution

<<< Supplementary Fig. 4, Related to Fig. 3. Positive control of GLOE-Seq, and genome-wide distribution of DNA breaks. (a) Upper panel: Sequence logo showing sequence conservation around the DNA breaks (dashed line) identified in naked DNA that, first, underwent blocking of existing (endogenous and handling-associated) breaks and, second, was treated with the nicking endonuclease Nb.BsrDI. Lower panel: Cumulative fraction of identified DNA breaks along the increasing distance from the 5' end of the Nb.BsrDI recognition pattern CATTGC. The non-zero distance from the 5' end of Nb.BsrDI recognition pattern corresponds to potential sloppiness (imperfect specificity) of the endonuclease. Precision, *i.e.*, the fraction of DNA breaks introduced by Nb.BsrDI, was estimated considering the distances 0 and 5 (82.46-84.25%). **(b)** Sensitivity estimated as the fraction of detected Nb.BsrDI recognition patterns CATTGC in the human reference genome. Assuming the endonuclease imperfect specificity and the distance smaller or equal to 5 (a), the sensitivity is 87.65%, whereas it is 87.32% when only the distance 0 is considered. The method can identify multiple breaks at one location (**Fig. 3a**), therefore, the number of identified DNA breaks in **a** is higher (around 20-fold) than the number of recognition patterns in the genome. **(c)** Genome-wide distribution of DNA breaks at 100-Kb resolution across cell lines with and without trabectedin exposure. Presented: single biological replicates. Gray: heterochromatin and short-arm gaps. Horizontal dashes: centromeres, whose data are not shown. DNA break count is defined in **Methods**. DNA break count values are capped at 4.44. **(d-e)** DNA-break counts are elevated following trabectedin treatment in TC-NER-proficient cells. **(d)** For each biological replicate, we summed DNA-break counts within chromosome 19 (upper panel) or the whole genome (lower panel). We show the min-max range and mean of this value across the replicates. *: the min-max ranges of 0 and 50 nM exposures do not overlap, indicating the specificity of trabectedin DNA-break induction. n=2 in U2OS WT and *CSB*-KO (except *CSB*-KO 0 nM with n=1), n=3 in the rest. **(e)** P-value of Mann-Whitney U test with the one-sided alternative hypothesis that the trabectedin-treatment-related distribution is stochastically greater than the control distribution. For each cell line, 50 and 0 nM drug exposure experiments with highest sequencing depth are compared. A star: p-val < 0.001 (solid horizontal line), a red circle: p-val < 0.05 (dashed line), a black circle: p-val > 0.05. Sample size for the test in chr19: n=558, in whole genome: n=28,513. **(f)** Means (bars) of Spearman correlation coefficient across pairs of biological replicates (markers), n=1 pair in U2OS WT and *CSB*-KO, n=3 in the rest. Source data, including Mann-Whitney U test statistics and exact p-values, are provided as a Source Data file.

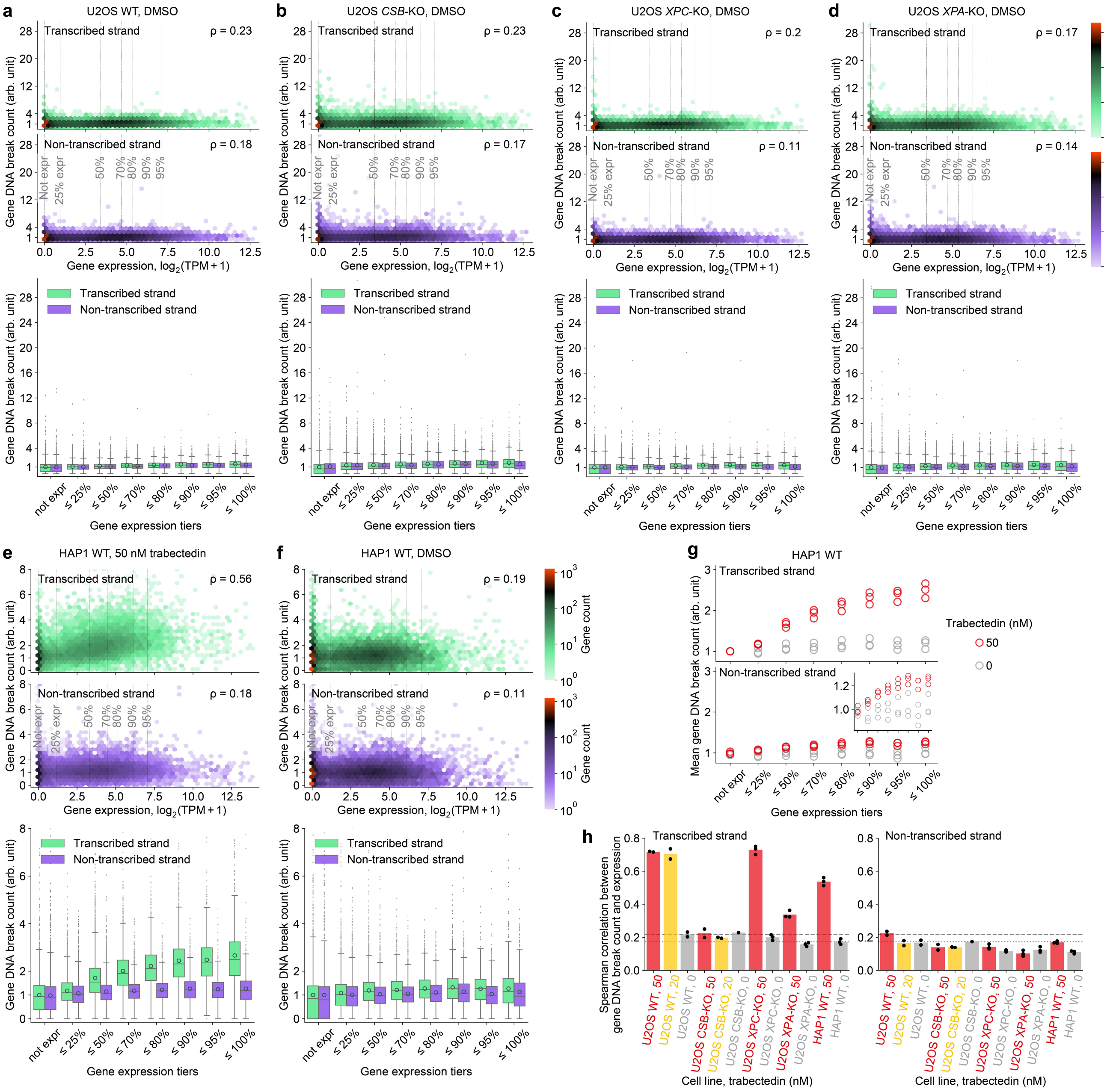

Supplementary Fig. 5, Related to Fig. 4. DNA break counts versus gene expression levels in trabectedin-untreated and treated U2OS and HAP1 cells. (a-d) DNA break count on the transcribed and non-transcribed strands of protein-coding genes in U2OS WT (a), *CSB*-KO (b), *XPC*-KO (c) and *XPA*-KO (d) after two-hour exposure to DMSO and subsequent two-hour recovery versus gene expression level in unexposed U2OS WT. **(e-f)** DNA break count on the transcribed and non-transcribed strands of protein-coding genes in HAP1 WT after two-hour exposure to trabectedin **(e)** or DMSO **(f)** and subsequent two-hour recovery versus gene expression level in unexposed HAP1 WT. **a-f**: The plots are built analogously to Fig. 4a-d. **(g)** Mean DNA break count of protein-coding genes in HAP1 WT after two-hour exposure to trabectedin or DMSO and subsequent two-hour recovery versus gene expression level in unexposed HAP1 WT. The plot is built analogously to Fig. 4e-h. **(h)** Correlation between gene

break count on an indicated strand and gene expression (n=16,740 genes) across cell lines and trabectedin exposure concentrations. Bar: mean across biological replicates shown as markers. Horizontal lines: mean correlation values for the transcribed strand in unexposed U2OS WT and HAP1 WT; these lines highlight that without chemical exposure, DNA break count correlates with gene expression stronger for the transcribed strand than for the non-transcribed one. Source data are provided as a Source Data file.

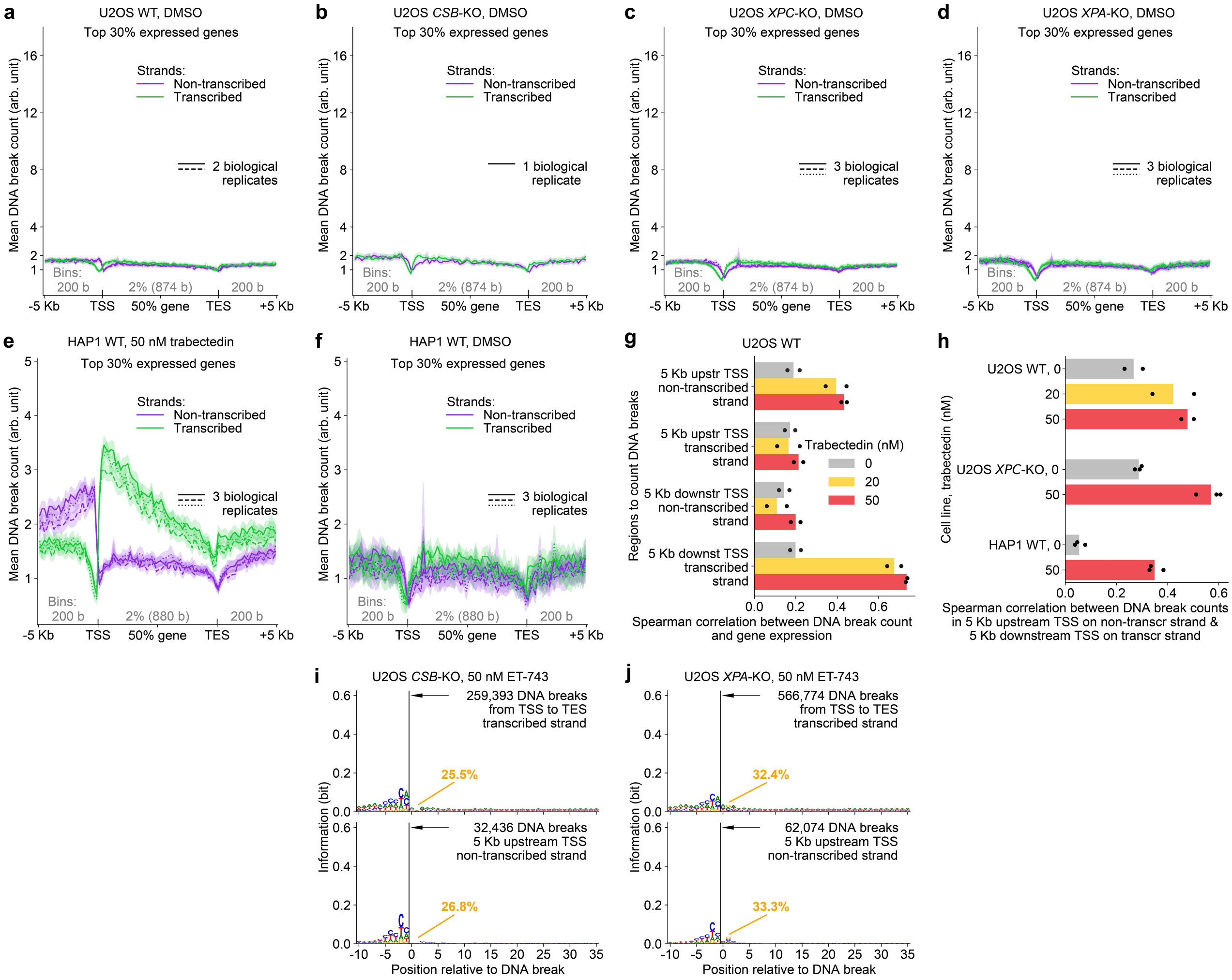

Supplementary Fig. 6, Related to Fig. 6. DNA-break profiles throughout genes in trabectedin-untreated and treated U2OS and HAP1 cells; correlation analysis of DNA breaks linked to divergent transcription; lack of sequence enrichment around DNA breaks in TC-NER-deficient cells. (a-d) Strand-specific profile of the mean DNA break count throughout the gene body and its upstream and downstream 5 kilobase regions in U2OS WT (a), *CSB*-KO (b), *XPC*-KO (c) and *XPA*-KO (d) after two-hour exposure to DMSO and subsequent two-hour recovery. n=4,425 genes considered. **(e-f)** Strand-specific profile of the mean DNA break count throughout the gene body and its upstream and downstream 5 kilobase regions in HAP WT after two-hour exposure to trabectedin I or DMSO (f) and subsequent two-hour recovery. n=3,994 genes considered. A-f: The plots are built analogously to Fig. 6a-d. **(g)** Correlation between DNA break count in the indicated regions and gene expression in U2OS WT exposed to 0, 20 and 50 nM trabectedin for two hours with subsequent two-hour recovery. **(h)** Correlation between DNA break counts along two branches of divergent transcription in TC-NER proficient cell lines. G-h: all protein-coding genes (n=16,740 genes) are considered, bars: means of biological replicates (markers, n=2 for each drug concentration in U2OS WT, n=3 for each drug concentration in the other two cell lines). **(i-j)** Sequence logos showing no sequence enrichment around DNA breaks in U2OS CSB-KO (i) and XPA-KO (j). The percentage of G at position 1 (+2 relative to the break)

is shown. The plots are built analogously to Fig. 6i-j. Source data are provided as a Source Data file.

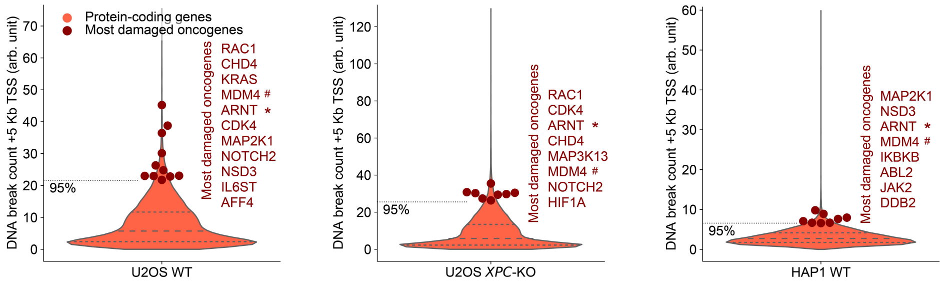

Supplementary Fig. 7, Related to Fig. 6. The distribution of gene DNA break counts in U2OS WT, U2OS *XPC*-KO and HAP1 WT across protein-coding genes. N=16,740 protein-coding genes. Most damaged oncogenes (higher than 95th percentile of the total distribution) are shown as markers and named in the order of descending DNA break count. #, *: the oncogenes found in the group of the most damaged genes across the three cell lines. Oncogene annotation: COSMIC Cancer Gene Census, tier 1, genes with documented activity relevant to cancer. Violin plots: the profiles show fitted density functions cropped at the minimal and maximal values; the internal horizontal lines show the upper quartile, median and lower quartile. DNA break data: gene DNA break counts were calculated within 5 kilobases downstream of the TSS (transcription start site) considering both strands; these counts were averaged across biological replicates (n=2 for U2OS WT, n=3 for the other two cell lines). Arb. unit: **Methods** describe DNA break count normalization. Source data are provided as a Source Data file.

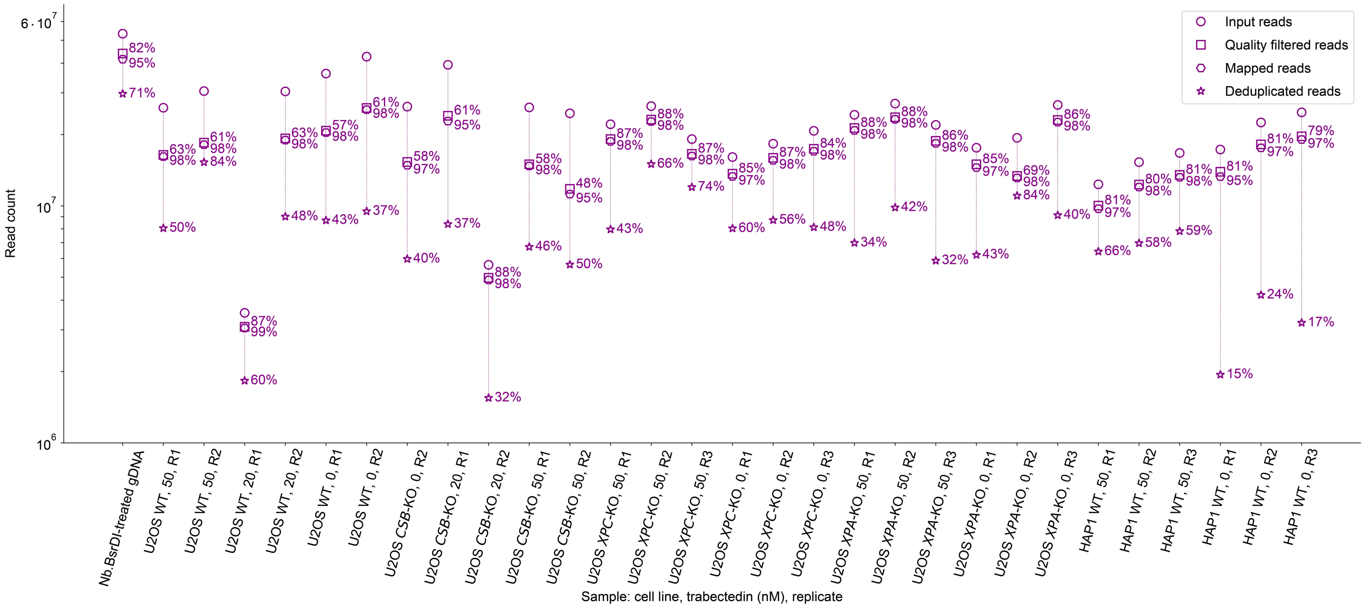

Supplementary Fig. 8, Related to Figs. 3-6. The evolution of read counts in individual samples throughout the steps of read processing. The indicated percentage is the fraction of reads remaining after the respective processing step. Mapping efficiency is on average 97% across the samples. Source data are provided as a Source Data file.
