# Supplementary material for: Trabectedin derails transcription-coupled nucleotide excision repair to induce DNA breaks in highly transcribed genes
Source: Nat Commun. 2024 Feb 15;15:1388. doi: 10.1038/s41467-024-45664-7 (PMC10869700; doi:10.1038/s41467-024-45664-7)
Supplement: Supplementary file 3 — Description of Additional Supplementary Files [file 41467_2024_45664_MOESM3_ESM.pdf]

Description of Additional Supplementary Files

File Name: Supplementary Data 1

Description: List of reagents, enzymes, kits, oligonucleotides, and other materials used for GLOE/TRABI-seq experiments.
