# Supplementary material for: Trabectedin derails transcription-coupled nucleotide excision repair to induce DNA breaks in highly transcribed genes
Source: Nat Commun. 2024 Feb 15;15:1388. doi: 10.1038/s41467-024-45664-7 (PMC10869700; doi:10.1038/s41467-024-45664-7)
Supplement: Supplementary file 5 — Reporting Summary [file 41467_2024_45664_MOESM5_ESM.pdf]

nature portfolio

Corresponding author(s): Shana J. Sturla, Orlando D. Schärer

Last updated by author(s): Jan 10, 2024

Reporting Summary

Nature Portfolio wishes to improve the reproducibility of the work that we publish. This form provides structure for consistency and transparency in reporting. For further information on Nature Portfolio policies, see our Editorial Policies and the Editorial Policy Checklist.

Statistics

For all statistical analyses, confirm that the following items are present in the figure legend, table legend, main text, or Methods section.

n/a	Confirmed	
☐	☒	The exact sample size (*n*) for each experimental group/condition, given as a discrete number and unit of measurement
☐	☒	A statement on whether measurements were taken from distinct samples or whether the same sample was measured repeatedly
☐	☒	The statistical test(s) used AND whether they are one- or two-sided<br>*Only common tests should be described solely by name; describe more complex techniques in the Methods section.*
☒	☐	A description of all covariates tested
☐	☒	A description of any assumptions or corrections, such as tests of normality and adjustment for multiple comparisons
☐	☒	A full description of the statistical parameters including central tendency (e.g. means) or other basic estimates (e.g. regression coefficient) AND variation (e.g. standard deviation) or associated estimates of uncertainty (e.g. confidence intervals)
☐	☒	For null hypothesis testing, the test statistic (e.g. *F*, *t*, *r*) with confidence intervals, effect sizes, degrees of freedom and *P* value noted<br>*Give P values as exact values whenever suitable.*
☒	☐	For Bayesian analysis, information on the choice of priors and Markov chain Monte Carlo settings
☒	☐	For hierarchical and complex designs, identification of the appropriate level for tests and full reporting of outcomes
☐	☒	Estimates of effect sizes (e.g. Cohen's *d*, Pearson's *r*), indicating how they were calculated

Our web collection on statistics for biologists contains articles on many of the points above.

Software and code

Policy information about availability of computer code

Data collection	Microscopy images were acquired using a fluorescence microscope (BX53, Olympus).<br>Western blot images were acquired using an automated imaging system (ChemiDoc touch imaging system, Bio-Rad Laboratories).<br>DNA libraries were sequenced on an Illumina NovaSeq 6000 with a single-read protocol and the read length of 101 bp (R1).
Data analysis	1) Analysis of Comet assay and survival data.<br>Microscopy images were analyzed in the Comet analysis software from Trevigen (Catalog # 4260-000-CS).<br>Statistical analyses were done using GraphPad Prism (version 9.0.0) and Microsoft Excel 365.<br>Graphs were plotted using GraphPad Prism (version 9.0.0).<br>2) Genome-scale data analysis.<br>The code for genome-scale data analysis required to generate respective figures is available at https://gitlab.ethz.ch/eth_toxlab/trabi-seq.<br>Sequencing read processing was implemented using FastQC/0.11.9, trimmomatic/0.38, bowtie2/2.3.5.1, umi_tools/1.1.2, samtools/1.12, bedtools2/2.29.2, custom Python/3.7.4 scripts using biopython/1.79, numpy/1.21.5 and pandas/0.25.1. The downstream analysis of DNA break data and their visualization were performed via custom Python/3.7.4 scripts, bedtools2/2.29.2 and Jupyter notebooks employing the modules numpy/1.19.2, scipy/1.6.3, pandas/1.1.3, biopython/1.79, logomaker/0.8, matplotlib/3.4.2 and seaborn/0.11.1 in Python/3.8.5 environment.

For manuscripts utilizing custom algorithms or software that are central to the research but not yet described in published literature, software must be made available to editors and reviewers. We strongly encourage code deposition in a community repository (e.g. GitHub). See the Nature Portfolio guidelines for submitting code & software for further information.

Data

Policy information about availability of data

All manuscripts must include a data availability statement. This statement should provide the following information, where applicable:

- Accession codes, unique identifiers, or web links for publicly available datasets
- A description of any restrictions on data availability
- For clinical datasets or third party data, please ensure that the statement adheres to our policy

The raw sequencing data and processed sequencing data (tsv-files with called DNA breaks) generated in this study have been deposited in the NCBI Gene Expression Omnibus (GEO) under accession code GSE245883 [https://www.ncbi.nlm.nih.gov/geo/query/acc.cgi?acc=GSE245883]. Source data, including the western blots in Supplementary Figs. 2a and 3a, are provided with this paper.
In the data analysis, we used the following external publicly available datasets. Human reference genome, GRCh38 [https://genome-idx.s3.amazonaws.com/bt/GRCh38_noalt_as.zip] (pre-built bowtie2 index). Transcript coordinates: GENCODE/V41/knownGene, retrieved from UCSC Table Browser. Canonical transcripts of genes: GENCODE/V41/knownCanonical, retrieved from UCSC Table Browser. Gene expression: DepMap Public 22Q2 [https://depmap.org/portal/download/all/?releasename=DepMap+Public+22Q2&filename=CCLE_expression_full.csv], the cell-line accession numbers ACH-000364 (U2OS WT) and ACH-002475 (HAP1 WT). Protein-coding genes: GENCODE/V41/knownToNextProt, retrieved from UCSC Table Browser. Coordinates of centromeres and gaps: retrieved from UCSC Table Browser for GRCh38. Chromatin accessibility and histone modification: from GEO [https://www.ncbi.nlm.nih.gov/geo/query/acc.cgi?acc=GSE87831], accession number GSE87831, specifically, GSE87831_DNase-Seq.r1.peaks.bed.gz, GSE87831_DNase-Seq.r2.peaks.bed.gz, GSE87831_H3K4me3.peaks.bed.gz (referred to as H3K4me3 [1] in Fig. 3c); from GEO [https://www.ncbi.nlm.nih.gov/geo/query/acc.cgi?acc=GSE44672], accession number GSE44672, specifically, GSM1356566_U2OS_H3K4me3.txt.gz (referred to as H3K4me3 [2] in Fig. 3c), GSM1356565_U2OS_H3K4me1.txt.gz, GSM1356567_U2OS_H3K27ac.txt.gz; genomic coordinates were converted from hg19 to hg38 via https://genome.ucsc.edu/cgi-bin/hgLiftOver. Oncogenes: COSMIC Cancer Gene Census [https://cancer.sanger.ac.uk/census] (downloaded on 15.05.2023).

Research involving human participants, their data, or biological material

Policy information about studies with human participants or human data. See also policy information about sex, gender (identity/presentation), and sexual orientation and race, ethnicity and racism.

Reporting on sex and gender	n/a
Reporting on race, ethnicity, or other socially relevant groupings	n/a
Population characteristics	n/a
Recruitment	n/a
Ethics oversight	n/a

Note that full information on the approval of the study protocol must also be provided in the manuscript.

Field-specific reporting

Please select the one below that is the best fit for your research. If you are not sure, read the appropriate sections before making your selection.

☒ Life sciences ☐ Behavioural & social sciences ☐ Ecological, evolutionary & environmental sciences

For a reference copy of the document with all sections, see nature.com/documents/nr-reporting-summary-flat.pdf

Life sciences study design

All studies must disclose on these points even when the disclosure is negative.

Sample size	Sample size determination was not based on any statistical method. For the various experimental methods, we chose sample sizes based on the technical challenges and the throughput of each assay. These selected sizes align with those in earlier studies.<br>For every comet chip assay, a minimum of 50 comets for each condition were evaluated to reach a conclusion (Nucleic Acids Res 48, e13 (2020)). For GLOE-Seq/TRABI-Seq experiments, the sample sizes were determined on the basis of the maximal throughput that can be achieved by an experimenter. Specifically, maximally 6 samples could be processed in parallel in the 10-day-long DNA-library-preparation protocol. This resulted in 6 samples for each of the following cell lines: U2OS WT (2x 50 nM drug, 2x 20 nM, 2x DMSO), U2OS XPC-KO (3x 50 nM drug, 3x DMSO), U2OS XPA-KO (3x 50 nM drug, 3x DMSO) and HAP1 WT (3x 50 nM drug, 3x DMSO), as wells as 5 samples for U2OS CSB-KO (2x 50 nM drug, 2x 20 nM, 1x DMSO). Three cell lines (U2OS WT, U2OS XPC-KO and HAP1 WT) are TC-NER-proficient and two cells lines (U2OS XPA-KO and U2OS CSB-KO) are TC-NER-deficient, which provides additional power for characterizing TC-NER involvement in the drug toxicity mechanism.
Data exclusions	Immunoblots with low signal-to-noise ratios that could not be quantified were excluded.<br>Preliminary data used to optimize the conditions for Comet Chip assays for each cell line were excluded.

For GLOE-Seq/TRABI-Seq data analysis, reads with low quality and Illumina adapter read-through were removed. The criteria for this exclusion and the counts of removed reads are provided in Methods and Supplementary Figure 8.

Replication

The number of repeated experiments is detailed in the manuscript's figure legends, in figures and Methods. All replication attempts were successful.

Randomization

Whole field of comet chip's microwells (96-well) were captured in fluorescence image analyses. All images captured from the Comet Chip assays were used and analyzed by the Comet analysis software from Trevigen (catalog # 4260-000-CS), negating the need for randomization in our study. For GLOE-Seq/TRABI-Seq experiments, cells that initiated a culture to be exposed to the drug and cells that initiated a culture to be exposed to the vehicle were split from a parent isogenic culture, therefore the cells were randomly distributed.

Blinding

There was no subjective allocation for any experiments, thus blinding was not required for this study.

Reporting for specific materials, systems and methods

We require information from authors about some types of materials, experimental systems and methods used in many studies. Here, indicate whether each material, system or method listed is relevant to your study. If you are not sure if a list item applies to your research, read the appropriate section before selecting a response.

Materials & experimental systems

n/a	Involved in the study
☐	☒ Antibodies
☐	☒ Eukaryotic cell lines
☒	☐ Palaeontology and archaeology
☒	☐ Animals and other organisms
☒	☐ Clinical data
☒	☐ Dual use research of concern
☒	☐ Plants

Methods

n/a	Involved in the study
☒	☐ ChIP-seq
☒	☐ Flow cytometry
☒	☐ MRI-based neuroimaging

Antibodies

Antibodies used

Antibodies were obtained from the following sources:
Mouse monoclonal anti-beta-Actin (BA3R) (WB dilution 1:10,000, Invtirogen, catalog # MA5-15739, RRID: AB_10979409)
Rabbit polyclonal anti-ERCC5/XPG (WB dilution 1:500, Bethyl, catalog # A301-484A, RRID: AB_999684)
Rabbit polyclonal anti-XPF (WB dilution 1:2000, Abcam, catalog # ab76948, RRID: AB_1524575)
Mouse monoclonal anti-XPF (WB dilution 1:200, Santa Cruz, catalog # sc-136153, RRID: AB_2098034)
Mouse monoclonal anti-ERCC1 (WB dilution 1:300, Santa Cruz Biotechnology, catalog # sc-17809, RRID: AB_2278023)
Goat anti-rabbit IgG, Polyclonal antibody, HRP-conjugate (WB dilution 1:2000, Enzo Life Sciences, catalog # ADI-SAB-300-J, RRID: AB_11179983)
Goat anti-mouse IgG F(ab')2, Polyclonal antibody, HRP-conjugate (WB dilution 1:2000, Enzo Life Sciences, catalog # ADI-SAB-100-J, RRID: AB_11179634)

Validation

The antibodies have been commercially obtained and were validated in multiple previous studies. The followings are Research Resource Identifiers (RRIDs) from Resource Identification Portal.
Mouse monoclonal anti-beta-Actin (BA3R) (RRID: AB_10979409, https://www.thermofisher.com/antibody/product/beta-Actin-Loading-Control-Antibody-clone-BA3R-Monoclonal/MA5-15739)
Rabbit polyclonal anti-ERCC5/XPG (RRID: AB_999684, https://www.thermofisher.com/antibody/product/ERCC5-XPG-Antibody-Polyclonal/A301-484A)
Rabbit polyclonal anti-XPF (RRID: AB_1524575, https://www.abcam.com/products/primary-antibodies/xpf-antibody-ab76948.html)
Mouse monoclonal anti-XPF (RRID: AB_2098034, https://www.scbt.com/p/xpf-antibody-3f2-3)
Mouse monoclonal anti-ERCC1 (RRID: AB_2278023, https://www.scbt.com/p/ercc1-antibody-d-10)
Goat anti-rabbit IgG, Polyclonal antibody, HRP-conjugate (RRID: AB_11179983, https://www.enzolifesciences.com/ADI-SAB-300/goat-anti-rabbit-igg-polyclonal-antibody-hrp-conjugate/)
Goat anti-mouse IgG F(ab')2, Polyclonal antibody, HRP-conjugate (RRID: AB_11179634, https://www.enzolifesciences.com/ADI-SAB-100/goat-anti-mouse-igg-f-ab-2-polyclonal-antibody-hrp-conjugate/)

Eukaryotic cell lines

Policy information about cell lines and Sex and Gender in Research

Cell line source(s)

HAP1 wild-type, XPC-, XPA-, CSB-, and XPG-KO cells were from Horizon Discovery.
HAP1 XPG-E791A, XPF-D687A, and ERCC1-KO cells were generated with CRISPR-Cas9 for this study.
XP2YO, XP2YO complemented with wild-type XPF or mutant XPF-D687A and XP2OS complemented with wild-type XPA were generated by lentiviral transfection (Staresincic, L. et al., 2009; Kim, M. et al., 2022).
XP3BR cells were from Kaoru Sugasawa (Kobe University).

XP3BR complemented with wild-type XPG or XPG-E791A were generated by lentiviral transfection for this study.
U2OS wild-type, XPC-, CSB-, and XPA-KO cells were from Martijn S. Luijsterburg (Leiden University Medical Center) (van der Weegen, Y. et al., 2020)
U2OS DDB2- and XPF-KO cells were from Hannes Lans, Jurgen A. Marteijn and Wim Vermeulen (Eramus University Medical Center) (Sabatella, M. et al., 2018; Ribeiro-Silva, C. et al., 2020).

Authentication	All cell lines were monitored using morphology and growth characteristics. Cellular morphology was daily examined. All mutant cell lines were validated by Western blot analysis and DNA sequencing.
Mycoplasma contamination	All cell lines were periodically tested for mycoplasma contamination and mycoplasma negative.
Commonly misidentified lines (See ICLAC register)	No commonly misidentified cell lines were used.
